# Loop-Mediated Isothermal Amplification Methods for Diagnosis of Bacterial Meningitis

**DOI:** 10.3389/fped.2018.00057

**Published:** 2018-03-12

**Authors:** Mitsuko Seki, Paul E. Kilgore, Eun Jin Kim, Makoto Ohnishi, Satoshi Hayakawa, Dong Wook Kim

**Affiliations:** ^1^Division of Microbiology, Department of Pathology and Microbiology, Nihon University School of Medicine, Tokyo, Japan; ^2^Department of Pharmacy Practice, Eugene Applebaum College of Pharmacy and Health Sciences, Wayne State University, Detroit, MI, United States; ^3^Department of Pharmacy, College of Pharmacy, Hanyang University, Ansan, South Korea; ^4^Institute of Pharmacological Research, Hanyang University, Ansan, South Korea; ^5^Department of Bacteriology I, National Institute of Infectious Diseases, Tokyo, Japan

**Keywords:** loop-mediated isothermal amplification, meningitis, cerebrospinal fluid, *Neisseria meningitidis*, *Haemophilus influenzae*, *Streptococcus pneumoniae*, serotype identification

## Abstract

The rapid, accurate, and efficient identification of an infectious disease is critical to ensure timely clinical treatment and prevention in public health settings. In 2015, meningitis caused by *Streptococcus pneumoniae, Haemophilus influenzae*, and *Neisseria meningitidis* was responsible for 379,200 (range: 322,700–444,700) deaths. Clinical features alone cannot determine whether bacterial meningitis is present; an analysis of cerebrospinal fluid (CSF) is essential. Loop-mediated isothermal amplification (LAMP) is a nucleic acid amplification method offering an alternative to polymerase chain reaction (PCR). LAMP-based assays for detection of three leading bacteria in CSF for diagnosis of meningitis have been established. The typing assays using LAMP for detection of meningococcal serogroups A, B, C, W, X, and Y as well as *H. influenzae* serotypes a, b, c, d, e, and f were launched. In comparative analysis of the meningitis pathogen assays, LAMP assays did not yield false negative results, and the detection rate of LAMP assays was superior compared with PCR or conventional culture methods. LAMP assays provide accurate and rapid test results to detect major bacterial meningitis pathogens. Accumulating evidence suggests that LAMP assays have the potential to provide urgently needed diagnostics for bacterial meningitis in resource-limited settings of both developed and developing countries.

## Introduction

Over the past several decades, the incidence of meningitis caused by *Streptococcus pneumoniae* and *Haemophilus influenzae* in children has declined due to widespread vaccination (1, 2). Recent success in the introduction of the meningococcal conjugate serogroup A vaccine has underscored the importance of continued clinical and epidemiologic surveillance for invasive meningococcal disease across countries where the vaccine has been deployed (3). At the same time, the incidence of invasive diseases due to bacterial strains that were not included in the vaccines has increased (2, 4, 5). Even with appropriate antimicrobial therapy, the burden of disease in adults and mortality both in adults and children remain high in several countries, particularly resource-limited countries (6).

Polymerase chain reaction (PCR)-based assays are relatively expensive and complex to perform in resource-limited laboratory settings. Loop-mediated isothermal amplification (LAMP) is a nucleic acid amplification method offering the rapid, accurate, and easy to use diagnosis of infectious diseases (7, 8). LAMP-based diagnostic assays for *S. pneumoniae, H. influenzae*, and *N. meningitidis* have been established (9–13). The typing assays using LAMP for the detection of *N. meningitidis* serogroups [A, B, C, W, X, and Y (14)] and *H. influenzae* serotypes [a, b, c, d, e, and f (15)] were released.

In 2015, meningitis caused by *S. pneumoniae, H. influenzae*, and *N. meningitidis* was responsible for 379,200 (range: 322,700–444,700) deaths (16). Clinically, the rapid, accurate, and efficient identification of an infectious agent is critical to ensure prompt, safe and effective therapy. Yet, clinical features alone cannot determine whether bacterial meningitis is present; an analysis of cerebrospinal fluid (CSF) is essential for diagnosis. Bacterial culture methods require a well-equipped laboratory with appropriate biosafety facilities, specialized bacterial culture media, and reagents. In developing countries, the accurate diagnosis of bacterial meningitis remains a challenge due to the limited availability of routine microbiology laboratory services (17). Moreover, because early empiric treatment with antibiotics is commonplace in many countries, bacterial growth in standard culture media may be inhibited by the antibiotics present in human clinical samples.

For decades, the lack of available diagnostic tests in developing countries has been associated with widespread empiric antibiotic treatment and rising antibiotic resistance. There is urgent need to develop and deploy effective diagnostic tests that meet the criteria needed to ensure global access to end-users in developed and developing countries. Despite technological innovations, low-cost, sensitive and specific diagnostic tests for major bacterial pathogens remain unavailable in many resource-limited countries. The goal of this report is to review rationale, strategy, and opportunities for application of LAMP diagnostic methods for bacterial meningitis.

## LAMP Specificity

The isolation and identification of pneumococcus are complicated by contamination with alpha-hemolytic streptococci (*Streptococcus mitis* and *Streptococcus oralis*) belonging to the normal flora. *S. mitis* and *S. oralis* are most closely related to pneumococcus on this ecological basis, and their 16S rRNA sequences share over 99% identity with those of pneumococcus, making it difficult for clinical laboratories to differentiate pneumococcus from other oral alpha-hemolytic streptococci (18). Such misidentification might affect diagnosis and treatment.

The development of PCR-based assays targeting the *ply* (19) and *lytA* (20) genes was an important milestone in the laboratory diagnosis of pneumococcus. However, *ply* and *lytA*, initially thought to be present only in *S. pneumoniae*, were reported in strains of *S. mitis* (18). PCR-based tests that target these genes might result in the over-detection of pneumococcus. In many cases, PCR or real-time PCR methods are still not appropriate for resource-limited settings due to their technical requirements and cost.

The detection of *S. pneumoniae* teichoic acid antigen in urine (Binax NOW^®^ *S. pneumoniae*; Alere International Ltd., Galway, Ireland) is an alternative to culture or PCR; however, its specificity is low in children because of false-positive results in healthy children carrying pneumococci and other closely related *Streptococcus* species (21).

The matrix-assisted laser desorption ionization–time of flight mass spectrometry (MALDI-TOF MS) technique has emerged as a clinical microbiology laboratories for the identification of a wide range of pathogens, however, the specificity of MALDI-TOF test for the differentiation of pneumococci from other mitis group streptococci is still low and often recognized false-positives (22). Moreover, its apparatus is too expensive and complex to use in developing countries.

The LAMP assay has high specificity because the LAMP reaction occurs only when all six to eight regions within a target DNA are recognized correctly by the primers (8). Thus, the higher analytical specificity of the LAMP assay compared to other methods, including PCR, has been recognized in the published literature (9, 11, 23). Using 25 clinically isolated streptococcal strains, a LAMP test targeting *lytA* succeeded in differentiating 4 *S. pneumoniae* strains from 21 other *Streptococcus* species (3 *S. oralis*, 17 *S. mitis*, and 1 *Streptococcus* species) known to carry *ply* and, *lytA* whereas PCR produced 4 false-positive results for *lytA* and 17 false-positive results for *ply* (11, 23).

To assess *H. influenzae* type b (Hib) carriage, laboratory facilities for the reliable cultivation of Hib and identification of the capsular polysaccharide by immunological techniques are necessary. However, capsular antigen serotyping may produce inconsistent results; for example, 68% of isolates identified as Hib by serotyping yielded false-positives by PCR (24). Thus, in developing countries, the accurate diagnosis of Hib remains a challenge. A LAMP primer set for detecting Hib has been established and was compared with conventional PCR for the detection of *bexA* (25) and nested PCR for the detection of six capsule types of Hib (26). Using 46 *H. influenzae* strains and 21 non-*H. influenzae* strains, both of the nested PCR and LAMP assays for Hib accurately discriminated standard reference Hib strains from non-Hib strains, whereas the slide agglutination serotyping test yielded one false-positive and two false-negative results (9).

The identification and serogrouping of *N. meningitidis* infection are important for disease control and vaccination strategies. However, *N. meningitidis* is still diagnosed using classical culture methods. *Neisseria meningitidis* is subdivided into 13 serogroups based on the capsular polysaccharide type (A, B, C, D, E, H, I, K, L, W, X, Y, and Z); however, most invasive meningococcal disease has been attributed to six serogroups (A, B, C, W, X, and Y) (27). Five of these serogroups (A, B, C, W, and Y) are targets in currently licensed meningococcal vaccines.

A rapid diagnostic test using an immunochromatographic method for the identification of *N. meningitidis* serogroups A, C, W, and Y utilizing monoclonal antibodies against the polysaccharides of these four serogroups has been developed and used in endemic regions of Africa (28). Several PCR-based methods for the diagnosis of *N. meningitidis* infection and serogrouping of the six major serogroups have been developed (29, 30). Using the LAMP technique, *N. meningitidis* detection (12) and specific identification of the six major serogroups (A, B, C, W, X, and Y) of *N. meningitidis* (14) have been established, and their specificities have been confirmed.

## LAMP Sensitivity

Loop-mediated isothermal amplification employs a DNA polymerase with strand-displacement activity, along with two inner primers (forward and backward inner primers) and two outer primers (F3 and B3) that recognize six separate regions within a target DNA sequence. This method utilizes a unique priming mechanism that yields specific DNA products in less time than PCR. Additional primers (i.e., loop primers: loop primer forward and loop primer backward) designed to anneal to the loop structure in LAMP can be used to accelerate the LAMP reaction, resulting in enhanced sensitivity, and loop primers are now commonly used in practical applications of LAMP (8).

Table 1 shows detection limits of LAMP and PCR assays detecting DNA of meningitis pathogens and using spiked CSF specimens. The pneumococcus LAMP method could detect as few as 10 copies of both purified DNA and spiked CSF specimens within 30 min with greater sensitivity than conventional PCR (11). The sensitivity of the LAMP method was comparable to that of real-time PCR targeting *lytA* (31). Hib LAMP also detects 10 copies of purified DNA in a 60-min reaction, reflecting a detection limit with a more than 100-fold greater sensitivity than both PCR for the detection of *bexA* and nested PCR for the detection of Hib (9). The meningococcus LAMP assay was more sensitive than the previously described PCR method. The detection limit of the LAMP assay was measured down to around 10 copies per reaction with a greater sensitivity than that of conventional PCR (10, 12). Using the DNA spike CSF specimens as a template, the detection limit of meningococcal PCR detection method was attenuated while that of LAMP method unchanged.

**Table 1:** Limits of bacterial DNA detection in LAMP and PCR assays for meningitis pathogens using spiked CSF specimens.

Bacteria/primer target	Detection limit (purified DNA)		Detection limit (spiked CSF)	
	PCR	LAMP	PCR	LAMP
*S. pneumoniae*/*lytA* (11)	10^4^ copies/reaction	10	10^4^	10
Hib/capsulation locus region II (9)	10^3^	10	10^3^	10
*N. meningitidis*/*ctrA* (12)	10^4^	10	10^5^	10
*N. meningitidis*/*ctrA* (10)	10^a^	6	NA	NA
*N. meningitidis*/*ctrB* (13)	NA	10^2^	NA	NA

*^a^Real-time PCR results*.

*NA, not available*.

To obtain sensitive reaction results from conventional PCR assays, a DNA extraction and purification step is required because biological fluids and substances contained in clinical samples inhibit reactions (32–34). This additional step makes it difficult to perform PCR as part of a bedside examination because the DNA extraction is a somewhat troublesome procedure requiring more than 30 min to complete as well as specific equipment and the added expense of a DNA extraction kit. LAMP exhibits less sensitivity to inhibitory substances such as serum, plasma, urine, and culture medium present in biological samples than PCR (35). In fact, the robustness of LAMP in the face of reaction inhibitors can contribute to saving the time and cost required for sample processing steps.

## LAMP Assay Evaluation using Clinical CSF Specimens

The suitability of LAMP methods for detecting bacterial meningitis in CSF was validated with up to 1,574 randomly selected CSF samples from children with suspected meningitis in Korea, China, and Vietnam collected between 1999 and 2002 in prospective studies of bacterial meningitis (36–38). For comparison, CSF specimens were also tested by conventional PCR and culture tests. The comparative analysis of the meningitis pathogen LAMP assays, the clinical sensitivity, specificity, positive predictive value, and negative predictive value of the LAMP assays were compared with the PCR or conventional culture methods (9, 11, 12).

The pneumococcus detection rates of the LAMP, PCR and culture methods from CSF were 31.1% (33/106), 17.0% (18/106), and 10.4% (11/106), respectively. The detection rates for Hib were 57.7% (30/52), 42.3% (22/52), and 40.4% (21/52), respectively. Those of meningococcus were 2.0% (31/1,574), 1.6% (25/1,574), and 0.2% (3/1,574), respectively. The detection rates of the LAMP method were substantially higher than those of PCR and culture tests, even though the method for extraction of DNA was only heating at 95°C for 2 min.

Table 2 lists the overall performances of the LAMP and PCR assays used to detect three meningitis bacteria. The sensitivity, specificity, and positive and negative predictive values for the pneumococcus LAMP assay were 100.0% (18/18), 83.0% (73/88), 54.5% (18/33), and 100.0% (73/73), respectively, relative to PCR for diagnosis of pneumococcus infection. The comparable values of the Hib LAMP assay were 100.0% (22/22), 73.3% (22/30), 73.3% (22/30), and 100.0% (22/22), respectively. Those of the meningococcus LAMP assay were 100.0% (25/25), 99.6% (1,543/1,549), 80.6% (25/31), and 100.0% (1,543/1,543), respectively.

**Table 2:** A comparative analysis of PCR and LAMP for detection of bacterial meningitis.

Bacteria/primer target	Method for DNA extraction	Source population	Sample amount	Detect. rate (%)LAMP/PCR	SS (%)	SP (%)	PPV (%)	NPV (%)
*S. pneumoniae*/*lytA* (11)	95°C, 2 min	China, Korea, Vietnam	5 µl	31.1 (33/106)/17.0 (18/106)	100.0 18/18	83.0 73/88	54.5 18/33	100.0 73/73
Hib/capsulation locus region II (9)		Vietnam	5 µl	57.7 (30/52)/42.3 (22/52)	100.0 22/22	73.3 22/30	73.3 22/30	100.0 22/22
*N. meningitidis*/*ctrA* (12)		China, Korea, Vietnam	2 µl	2.0 (31/1,574)/1.6 (25/1,574)	100.0 25/25	99.6 1,543/1,549	80.6 25/31	100.0 1,543/1,543

*SS, sensitivity; SP, specificity; PPV, positive predictive value; NPV, negative predictive value*.

It seems that in clinical CSF specimens, these LAMP methods have almost perfect clinical sensitivity and negative predictive value with high clinical specificity and positive predictive value for the detection of the targeted bacterial meningitis pathogens. Moreover, the DNA extraction from CSF specimens can be easily achieved by heating to 95°C for 2 min.

In the United Kingdom, a bedside meningococcal LAMP test was determined to have a positive predictive value of 100% and a negative predictive value of 97%, while their samples included blood or combined nasal and throat swabs (39).

## Perspectives

Loop-mediated isothermal amplification methods for serogrouping meningococcus and serotyping *Haemophilus influenzae* have been established (9, 14, 15). However, LAMP methods for pneumococcus serotyping have not yet been available, and some studies have reported the incidence of invasive diseases due to pneumococcal strains that were not included in the vaccines (2, 40, 41). Pneumococcus has a highly diverse polysaccharide capsule, containing more than 90 different serotypes. The distribution of those serotypes varies geographically (42, 43). The available pneumococcal conjugate vaccines were designed to provide immunity against the most prevalent invasive serotypes worldwide (42). Understanding the geographic distribution and shifts in prevalence over time of every serotype is important for optimizing vaccine design and assessing the postvaccine introduction impact on disease burden. For the diagnosis of pneumococcal serotypes, LAMP tests that can generate multiple results simultaneously (multiplex format) would be advantageous.

Loop-mediated isothermal amplification reactions require separate reaction tubes for each pathogen or each target gene because test positivity is confirmed by the observation of turbidity in each reaction tube. The μTAS (micro Total Analysis Systems) or Lab on a Chip are next-generation LAMP detection devices (44) that enable the detection of multiple LAMP results simultaneously in a single microplate. Such a development has already started (45).

Because of the robustness and one-step reaction features of the LAMP assay (46), it could be easily applied to such next-generation reaction devices. In this case, a dried formulation of LAMP reagents (44) or a simple visual detection technique (7, 47) should be useful in developing LAMP tests capable of generating multiple results simultaneously. A multiple-LAMP assay for the diagnosis of bacterial meningitis could be applied to clinics in resource-limited settings. In this case, prospective studies of the LAMP assay should be done using clinical specimens and compare with conventional bacterial culture, antigen detection, PCR, etc.

## Clinical Application

Clinical diagnosis of acute and potentially fatal infectious diseases remains a major priority in developed and developing countries. While a host of new and relatively expensive diagnostic platforms are in clinical use and development, there remains a need for lower cost meningitis diagnostic tests that can be performed by laboratory technicians with limited training and time. As hospitals seek to contain rising healthcare costs, LAMP assays for meningitis offer rapid, accurate, and easy to use detection of bacterial DNA that can be completed in a short time compared with bacterial culture. LAMP assays for the diagnosis of meningitis also fulfill other key requirements of tests to detect dangerous pathogens including the ability to be completed safely in standard clinical laboratory facilities present in the majority of tertiary care and community hospitals around the world.

Presently, several manufacturers have successfully launched validated LAMP-based diagnostic tests (44). In all cases, the diagnostic test manufacturers have licensed the LAMP technology with royalties going to patent holders in Japan. In coming years (the end of 2018), the primary LAMP technology patent is expected to expire. When this occurs, LAMP technology may be accessible in a broad range of healthcare environments across several regions worldwide. Expanding LAMP technology access across low- and middle-income countries may also be vital to improving diagnostic capacity, supporting prudent antibiotic usage and combating antimicrobial resistance. Deployment of LAMP assays offers the potential to provide urgently needed diagnostics for bacterial meningitis in resource-limited countries where there is a need for accurate case diagnosis to guide therapy on the frontline of patient care.

## Public Health Perspectives

The rapidly evolving field of diagnostic testing has improved the availability of rapid diagnostic point-of-care (POC) tests that include assays that can be widely distributed and require a limited amount of training by end-users. For example, antigen detection POC tests that use urine specimens for testing have improved the detection of bacterial infections caused by *S. pneumoniae*—particularly in institutions where microbiologic culture methods may not be available (48). As molecular POC diagnostics such as LAMP assays become more widely available, their usage has the potential to improve public health surveillance and outbreak detection. Ultimately, the use of such tests can encourage more judicious use of antibiotics (with the potential for helping to reduce antimicrobial resistance) and improve control of diseases that threaten populations in developing and developed countries. Future research will be needed to identify how and where countries can best deploy LAMP POC tests in health systems to maximize their benefit to public health.

## Author Contributions

PK, DK, MS, MO, and SH contributed the conception of this manuscript; PK, DK, MS, and SH drafted the manuscript; and EK and MO did the critical review and approved the manuscript.

## Conflict of Interest Statement

The authors declare that the research was conducted in the absence of any commercial or financial relationships that could be construed as a potential conflict of interest.
